# Does a sense of gratitude protect against empathy loss in medical students? An exploratory study

**DOI:** 10.3205/zma001553

**Published:** 2022-07-15

**Authors:** Claire Elisabeth Vogel, Claudia Kiessling, Martin R. Fischer, Tanja Graupe

**Affiliations:** 1 Klinikum Landkreis Erding, Abteilung für Unfallchirurgie und Orthopädie, Erding, Germany; 2 Private Universität Witten/Herdecke gGmbH, Lehrstuhl für die Ausbildung personaler und interpersonaler Kompetenzen im Gesundheitswesen, Witten, Germany; 3 LMU Klinikum, LMU München, Institut für Didaktik und Ausbildungsforschung in der Medizin (DAM), München, Germany

**Keywords:** empathy, gratitude, protective factors, communicative skills, medical studies

## Abstract

**Introduction:**

The promotion of physicians’ empathy (PE) skills in medical school plays a central role in physician-patient communication. However, a significant decline in empathy among medical students during their training has been repeatedly reported. Gratitude could be a possible protective factor for PE. However, as some students do not seem to be affected by this empathy loss, this study explores the relationship between gratitude and PE.

**Methods:**

Using validated questionnaires (JSPE-S, IRI and GQ-6), 88 medical students at LMU München evaluated their self-assessed PE and gratitude. In addition, they went through four OSCE stations focusing on general medicine, in which their empathy and communication skills were assessed by simulated patients (SP) and by an assessor using the Berlin Global Rating. Correlations were analysed using Pearson's correlation coefficient and gender differences were analysed using Mann-Whitney U-tests.

**Results:**

In the self-assessment, there was a significant, moderate correlation between students' attitude towards empathy (JSPE-S) and their gratitude (GQ-6) and a weak correlation between the IRI subscale “Empathy” and the GQ-6. In terms of the performance-based assessment, there were also weak correlations between PE or communication skills and gratitude. There were no gender-specific differences in the gratitude of the students.

**Conclusion:**

We were able to demonstrate a correlational relationship between gratitude and empathy in medical students. Whether gratitude acts causally as a protective or supportive factor for empathy remains open. A causal relationship of gratitude to empathy should therefore be examined in a prospective study design.

## Zusammenfassung

**Zielsetzung:**

Die Förderung ärztlicher Empathiefähigkeit (ÄE) im Medizinstudium spielt eine zentrale Rolle für die Arzt-Patienten-Kommunikation. Jedoch fand sich bei Medizinstudierenden im Verlauf ihrer Ausbildung mehrfach ein signifikanter Empathierückgang. Dankbarkeitsempfinden (DE) könnte ein möglicher protektiver Faktor für ÄE sein. Da einige Studierende allerdings nicht von diesem Empathieverlust betroffen zu sein scheinen, erforscht diese Studie den Zusammenhang zwischen DE und ÄE.

**Methodik:**

Anhand von validierten Fragebögen (JSPE-S, IRI und GQ-6) wurde bei 88 Medizinstudierenden der LMU München ihre selbsteingeschätzte ÄE und ihr DE erhoben. Zusätzlich durchliefen sie vier allgemeinmedizinische OSCE-Stationen, in denen eine Einschätzung ihrer Empathie und kommunikativen Fähigkeiten durch Simulationspatient*innen (SP) sowie durch eine Beurteilerin mittels Berliner Global Rating erfolgte. Korrelationen wurden mittels Pearsons Korrelationskoeffizienten und geschlechtsspezifische Unterschiede mittels Mann-Whitney-U-Tests analysiert.

**Ergebnisse:**

In der Selbsteinschätzung zeigte sich zwischen der Einstellung der Studierenden zur Empathie (JSPE-S) und ihrem DE (GQ-6) ein signifikanter, mittelstarker Zusammenhang und ein schwacher Zusammenhang zwischen der IRI Subskala „Empathie“ und dem GQ-6. Bezüglich der Fremdeinschätzung zeigten sich zwischen ÄE bzw. kommunikativen Fähigkeiten und DE ebenfalls schwache Zusammenhänge. Es zeigten sich keine geschlechtsspezifischen Unterschiede bzgl. des DE der Studierenden.

**Schlussfolgerung:**

Wir konnten einen korrelationalen Zusammenhang zwischen DE und ÄE bei Medizinstudierenden nachweisen. Ob das DE kausal als protektiver oder unterstützender Faktor für ÄE wirkt, bleibt offen. Eine ursächliche Beziehung von DE zu ÄE sollte deshalb in einem prospektiven Studiendesign überprüft werden.

## 1. Introduction

Good communication skills are one of the core competencies of medical work, enable skillful handling of patients’ wishes, expectations and feelings and contribute to improving the quality of care [1], [2], [3], [4]. In this context, the ability of doctors to empathise plays a central role, as it promotes patients' satisfaction and adherence [4], [5], [6]. At the same time, it indirectly increases patients' education about their disease and treatment options and reduces their emotional burden [7]. In addition, medical empathy increases the willingness of the interlocutor to report symptoms and fears, which facilitates a targeted anamnesis and thus also optimises the diagnostic accuracy of those treating the patient [8], [9], [10]. 

Mercer and Reynolds conceptualised physician empathy as a multi-dimensional, multi-phase concept that has moral, emotional, cognitive and behavioural components [11] which entails to put oneself in patients' situations, experiences, feelings and perspectives [11]. It does not require a need to feel the other’s suffering on an emotional level [11], [12], [13], as this would lead to overidentification and a blurring of professional boundaries [11], [13], [14]. After an extensive literature review, Hojat and LaNoue define medical empathy as follows: “(...) empathy in the context of medical education and patient care was defined as a predominantly cognitive (as opposed to affective or emotional) attribute that involves an understanding (as opposed to feeling) of patients' experiences, concerns, and perspectives combined with a capacity to communicate this understanding and an intention to help by preventing and alleviating pain and suffering.” ([15], p.74).

The training of communicative and social skills has found its way into medical studies worldwide in recent decades [16], [17], [18]. The trainability of communicative and social skills, including empathy, has been demonstrated in a large number of studies [18], [19], [20], [21]. However, it has also been shown in a number of international studies that in the course of their training medical students seem to experience a significant decline in their empathy skills, especially when they enter the clinical phase [1], [22], [23], [24], [25], [26], [27]. Some studies have linked this loss of empathy to distress, which manifests itself in the form of burnout, stress, lack of sleep, low well-being, reduced quality of life or depression [28], [29], [30], [31], [32], [33], [34], [35]. The perceived workload and professional exhaustion of introverted and neurotic physicians was found to be higher than that of extroverted and less neurotic colleagues [36]. However, the loss of empathy continues not only in medical training but also among physicians who are already practising, mainly due to the above-mentioned aspects of distress, especially caused by time and performance pressure [28], [30]. The constant dichotomy between empathetically responding to the individual and, at the same time, a high clinical workload, in the absence of positive role models, has an impact not only on doctors' empathy [28], [29], [37] but also on the quality of care [28], [38], [39].

Despite various negative influencing factors, Hojat et al. were able to show that a notable proportion of medical students succeed in retaining their empathy skills over the entire course of their studies [35]. The results of the scoping review by Ferreira-Valente et al., in which no clear trend in the development of empathy during medical school could be demonstrated [40], are an indirect indication that, as Hojat et al. have already postulated, there are so-called “protective factors” that prevent students from losing empathy [35]. The question therefore arises as to what these protective factors are and to what extent they can be trained. 

Whereas the above-mentioned distress leads to a loss of empathy, good social support [41] as well as a high level of personal well-being [31], [32] and high intrinsic motivation [42] have a positive effect on physician empathy. It would be desirable to identify an overarching factor that not only promotes empathy but also counteracts negative factors influencing empathy. Gratitude could play a key role here, since gratitude, as a so-called prosocial ability of an individual, brings with it many positive characteristics [43] which could protect and promote empathy. Gratitude is the appreciation of a perceived personal benefit resulting from the action of another (human, non-human, natural or supernatural object) [44]. This personal benefit does not necessarily have to be earned by the recipient, but must explicitly intended for them, albeit in a metaphorical way [44]. Various studies have shown a positive influence of gratitude on well-being [45], [46], [47], [48], [49], sleep quality and duration [45], [50], social support [51], [52] and levels of depression and stress [48], [49], [51]. Furthermore, feelings of gratitude evoke prosocial behaviour and thus promote social relationships [52], [53], [54], [55]. McCullough et al. showed that grateful subjects were perceived as more emotionally helpful by those close to them [48]. The grateful subjects also rated themselves as more empathic, more extroverted and less neurotic compared to the less grateful subjects [48]. Since gratitude appears to have a positive influence on various factors that promote physician empathy, the aim of this study was to investigate the extent to which there is a connection between medical students' personal feeling of gratitude and their attitudes towards empathy or their empathic behaviour towards simulated patients in an OSCE.

## 2. Methods

### 2.1. Setting 

The overall aim of the study, of which the research project presented here was a defined sub-study, was on the one hand to develop different methods of testing communicative competences of medical students and on the other hand to investigate the correlations between knowledge, attitudes and behaviour in relation to dealing with emotions. The sub-study presented here dealt with the possible connection between gratitude and empathy of medical students from pre-clinical and clinical semesters at the Medical Faculty of the Ludwig-Maximilians-Universität München.

Students were invited to take part in an OSCE with four stations and a video-based Situational Judgement Test (SJT) on dealing with emotions as part of a laboratory study. In addition, the students filled out a questionnaire. Participation was voluntary and anonymous. The students received a voucher for 25 euros for their participation. The test development of the SJT was carried out by Graupe et al. [56] and the development of the OSCE was supported by Giemsa et al. [57] described elsewhere.

#### 2.2. Instruments

Medical students’ self-perceived gratitude was assessed using the Gratitude Questionnaire (GQ-6), which maps the individual facets of gratitude (intensity, frequency, span and density) without subscales based on a 7-point Likert scale from 1=“strongly disagree” to 7=“strongly agree” [48]. With a total of six items, the GQ-6 has a good internal reliability (Cronbach’s alpha=0.82) [48] and was used in this study in the German translation of Proyer (Cronbach’s alpha=0.69) [58].

Medical students' specific attitudes towards empathy as a cognitive construct were measured using the Jefferson Scale of Empathy – Student Version (JSPE-S) [59], [60], [61] or how relevant physician empathy is in the physician-patient relationship [62]. The JSPE-S consists of 20 items and is answered with a seven-point Likert scale from 1=“strongly disagree” to 7=“strongly agree” [63], [64]. The German translation of the JSPE-S used here has good internal reliability (Cronbach's alpha=0.82) [61]. 

In addition to the JSPE-S, the Interpersonal Reactivity Index (IRI) was used [65], which measures the cognitive and affective components of empathy on the basis of four subscales, independent of the target group [62]. The four subscales: Perspective Taking (PT), Fantasy (FS), Empathic Concern (EC) and Personal Distress (PD) are assessed with seven items each on a five-point Likert scale ranging from 1=“does not describe me well” to 5=“describes me very well” [65], [66]. According to Davis, the “Empathic Concern” subscale represents an emotional aspect of empathy [65]. The IRI scale has also been shown to have good psychometric properties [65], [66]. It was used in this study in the German version by Neumann et al [62]. 

The OSCE designed for the overall project consisted of four stations with typical scenarios from the general medical setting: a patient with a headache (KS), a patient with heart palpitations (HS) and the wish to find out more about check-up-35, a patient after a stay in hospital (KH) with a discharge letter and new medication, and a patient with insulin-dependent diabetes mellitus (DM). All simulated conversational interventions were video-recorded. The Berlin Global Rating (BGR) was used as a measuring instrument to assess the empathic behaviour and communication skills of the medical students [67]. The BGR was filled out by a rater after the OSCE using the video recordings as well as by the simulated patients (SP) directly after the ward. The BGR consists of four items that are rated on a five-point scale from 1=incompetent behaviour to 5=competent behaviour. The four items cover dealing with feelings and concerns (“Empathy”), structuring the conversation, verbal and non-verbal expression [67]. The SP were briefed and trained with the BGR as part of a two-hour role training. In an additional item, all SP assessed their overall impression of the test person using the question: “Imagine that this student is a practicing physician. Would you go to this student as a patient?”. This additional item was rated from 1=“I cannot imagine” to 5=“I can imagine well”. The rater (observer) was trained in separate rater training sessions:

1. Coding of a video with subsequent discussion and creation of a coding protocol with four raters;
2. parallel encoding of nine videos;
3. estimation of assessment agreement using Spearman's rho;
4. joint discussion of differences and consensus building;
5. re-coding of ten videos in parallel and checking of judgement agreement (Spearman's rho>.79 for all four raters).

The rater then coded all the remaining videos. 

#### 2.3. Data analysis

Data analysis was carried out with IBM Statistics SPSS version 21. Inverse formulated items were recoded after data entry. The loss of information due to incorrect or missing values was assessed to be low with an almost complete data set. Due to low internal consistency (Cronbach's alpha=0.27), the sum score for the BGR item “Empathy” was removed from further analyses of the SP assessment, but retained in the rater assessment (Cronbach's alpha=0.97) (see table 1 (Tab. 1)). For the analysis of each item, the mean and standard deviation (SD) were determined and their distributional properties were assessed using kurtosis and skewness. The internal consistency of each scale was checked using Cronbach's alpha. Pearson's bivariate correlations (r) were calculated with the data collected from the GQ-6, JSPE-S and IRI questionnaires and the BGR. The error probability was defined as 5% and the correlation coefficients were interpreted according to Cohen's template [68]. Any differences in gratitude between men and women were tested using the Mann-Whitney U test due to the unequal group sizes. 

## 3. Results

### 3.1. Sample

A total of 88 medical students took part in the study, 65 women (74%) and 20 men (23%). Three subjects (3%) did not indicate their gender. The age range was 18-42 years (median: 23 years). Thirty-three subjects (37.5%) came from the preclinical phase, 51 students (58%) from the clinical phase and 4 students (5%) from the practical year (PJ). For one test person, the entire SP assessment was missing, which is why this was removed from the analyses (n=87). With regard to gender, there were no significant differences in self-assessed gratitude and self-assessed empathy as measured by JSPE-S (women MW=36.9; men MW=36.3; p=.250 and women MW=118.2; men MW=13.2; p=.109, respectively). For the IRI subscales, there was a significant difference in relation to gender for the scales Empathic Concern (females MW=21.3; males MW=19.0; p=.005) and Fantasy (females MW=19.6; males MW=14.4; p=.001). The descriptive results of the scales used as well as the students’ results in the OSCE with the different perspectives “simulated patients” and “rater” are shown in table 1 (Tab. 1).

#### 3.2. To what extent does the self-perceived gratitude level of medical students correlate with their self-assessed attitude towards empathy? (first person perspective)

There was a moderate correlation between the GQ-6 and JSPE-S questionnaire (r=0.32; p=.003). The subscale “Empathic Concern” of the IRI questionnaire and the self-evaluated gratitude of the medical students showed a weak correlation (r=0.22; p=.043) (see table 2 (Tab. 2)).

#### 3.3. To what extent is there a positive correlation between the self-perceived gratitude of medical students and their externally assessed ability to empathize by ...

##### 3.3.1. Simulated patients (second person perspective)

There was a weak correlation (r=0.23; p=.030) between the SP’ BGR sum score and the subjects' self-evaluated gratitude. There was a borderline moderate correlation (r=0.29; p=.008) between the GQ-6 scale and the overall impression assessed by the SP (see table 2 (Tab. 2)).

##### 3.3.2. Rater (third person perspective)

Regarding the rater perspective, there was a weak correlation between the BGR sum score and the GQ-6 scale as well as between the BGR item “Empathy” and the GQ-6 scale (r=0.23; p=.029 and r=0.26; p=.015, respectively) (see table 2 (Tab. 2)).

## 4. Discussion

In line with the hypothesis, the present study was the first in German-speaking countries to demonstrate a moderate relationship between medical students' gratitude and their attitude towards empathy using the GQ-6 and JSPE-S self-assessment questionnaires. Comparable studies were able to demonstrate a significant relationship between the GQ-6 and the IRI subscale “Empathic Concern” [48], [69]. In our study, too, the correlation between these two scales was comparable as in McCullough et al. [48] weak. The BGR used by the SP and one rater showed a weak correlation between the medical students’ gratitude and their communication skills, which includes empathy, in both the second and third perspectives. In addition, McCullough et al. found a significant, weak positive relationship between self-evaluated empathy and gratitude assessed by others in their study with psychology students [48]. Furthermore, after their confirmatory factor analysis, the authors concluded that happiness, vitality, life satisfaction, optimism, and hope are related but not equivalent to gratitude [48]. With the correlations presented in this study, it can now be added that gratitude and empathy, or gratitude and communication skills, are related but can be distinguished from one other. 

Since our project is a correlational study, the question remains open whether gratitude causally influences empathy or vice versa. Following the theory of Wood et al. in which gratitude is seen in an interactive mutually reinforcing spiral with well-being and social relationships [43], gratitude could be an important resource and protective factor for empathy and vice versa, an indispensable prerequisite for gratitude. However, as critically noted by Wood et al, most scientific work on the subject of gratitude is based on correlations [43] which is why the results reported here are more of an exploratory nature. In order to investigate the extent to which gratitude remains stable over a longer period of time and to what extent it can be promoted through training, further data is required within the framework of prospective studies.

With regard to gender, there was no significant difference in the gratitude of the female and male candidates participating in this experiment. Other studies with non-medical person collectives regularly attribute a generally higher capacity for gratitude to women than to men [70], [71], [72]. Kashdan et al. [72] postulate that women express their feelings more often in order to benefit from advantages over their male colleagues. In addition, women could generally use their emotional intelligence to bind their counterpart, from whom they expect emotional or concrete support, to them by expressing gratitude. A limitation of our study is the relatively small group of men who participated in the study. Since a balanced gender distribution is difficult to realise given the predominant female quota of German medical students [73], attention should be paid to a larger test collective overall in subsequent studies in order to ensure a sufficient number of male participants and to be able to identify possible gender-specific differences. 

The additional question that the SP answered, namely whether they would go to the student as a patient, brought interesting results. According to this, the SP would prefer students with a higher level of gratitude in their future choice of physician. Gratitude also seems to have an indirectly supportive effect on the physician-patient relationship through its positive influence on empathy. But how does the medical students' gratitude directly promote the SP' trust in them, even more so when the interaction only took place within a ten-minute OSCE? Various studies have shown grateful people to have strong communicative and social skills: they are more empathetic, more extroverted, more stress resistant, more relaxed, less depressed, they show a high level of well-being and, above all, they are motivated to do favours [45], [46], [47], [48], [51], [52]. These effects of gratitude might have generated an overall confidence-inspiring image of the more grateful students among the SP and moved them to a positive evaluation of the additional question. 

A limitation of the study was the relatively short training of the SP on the use of the BGR. The SP' assessment of the students was significantly more positive than that of the more intensively trained rater. However, the strength of the correlation with the subjective feeling of gratitude was comparable for the SP and the rater.

In order to be able to test a direct influence of gratitude on the physician-patient relationship, the causality to empathy and the temporal stability of the construct, it would have been desirable to capture not only empathy but also gratitude from several perspectives, as recommended by Emmons et al. [74]. Here, additional behavioural measurement methods would be necessary to statistically better validate even subtle differences and to relativise the factor of social desirability in the self-assessment. 

Besides gratitude, other factors such as social support or other personality traits could also have a beneficial effect on physician empathy or at least prevent its loss. In the survey by Ahrweiler et al., extracurricular activities, characterised by personal or guided reflection, active self-development and non-medical experiences were mentioned as further empathy promoters [28]]. Further investigations that shed light on these and other factors and their protective and supportive influence on empathy would be desirable.

## 5. Conclusions

Gratitude on the part of the practitioners has a positive effect on the student-(simulated) patient relationship. In addition, the gratitude of medical students in this scientific work was shown to be independent of gender.

We found a significant correlation between gratitude and the ability to empathise in medical students. The extent to which gratitude can assume a temporally stable protective function for the preservation of empathy must be further clarified in future controlled prospective studies.

## Acknowledgement

The authors would like to thank all students of the Medical Faculty of LMU München who participated in this study. Thanks are also due to the simulated patients for accompanying the OSCE stations and the external evaluation of the students in the second perspective. Special thanks go to Clara Wübbolding and Katharina Schäfer for evaluating the test participants using the video rating and to Nurith Epstein for critically reviewing the manuscript. 

## Competing interests

The authors declare that they have no competing interests. 

## Figures and Tables

**Table 1:**
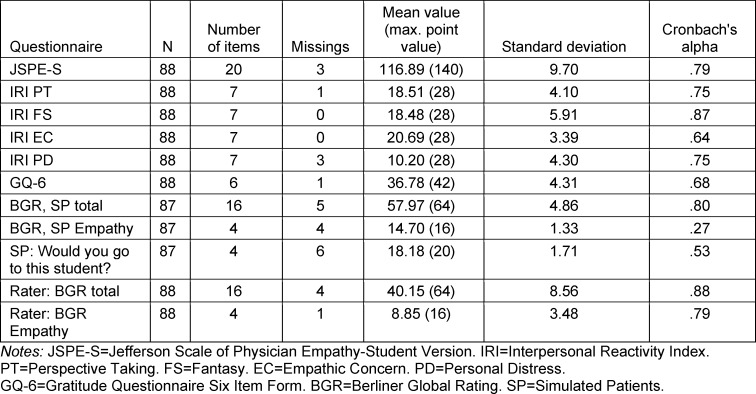
Item scale statistics of the questionnaires and measuring instruments used

**Table 2:**
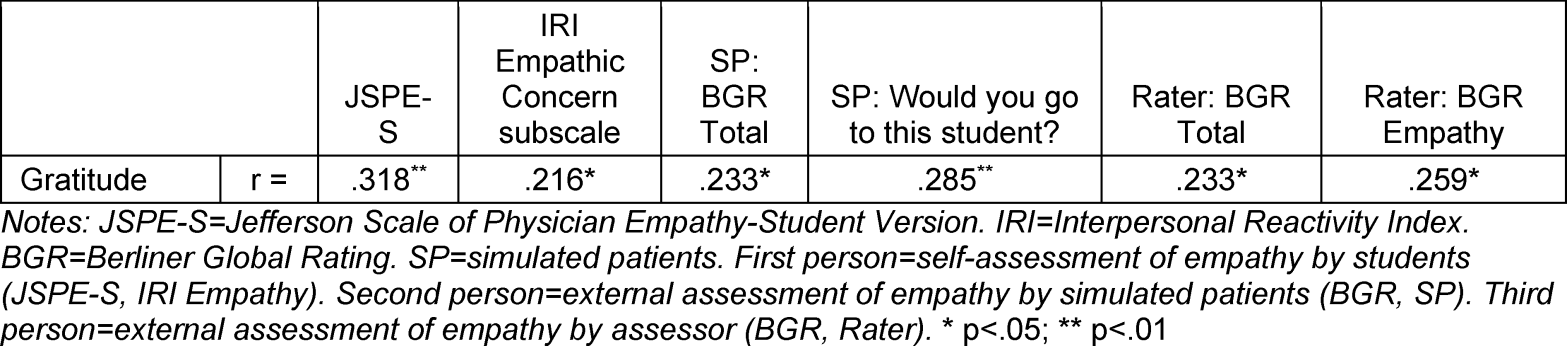
Bivariate correlation coefficients between gratitude and the first person, second person and third person assessment of empathy
